# Understanding Why Older Adults With Type 2 Diabetes Join Diabetes Online Communities: Semantic Network Analyses

**DOI:** 10.2196/10649

**Published:** 2018-06-28

**Authors:** Jakeem Amir Lewis, Perry M Gee, Chia-Ling Lynn Ho, Lisa M Soederberg Miller

**Affiliations:** ^1^ Department of Human Ecology University of California, Davis Davis, CA United States; ^2^ Nursing Research and Analytics Dignity Health San Francisco, CA United States; ^3^ College of Nursing University of Utah Salt Lake City, UT United States; ^4^ Department of Communication University of California, Davis Davis, CA United States

**Keywords:** online community, diabetes, health information, health support, chronic health difficulty, self-management, social support

## Abstract

**Background:**

As individuals age, chronic health difficulties may disrupt physical and social well-being. Individuals can turn to online communities to interact with similar peers, which may help buffer negative effects resulting from health difficulties.

**Objective:**

This study investigated the reasons that older adults join a diabetes online community to better understand the specific resources that are being sought.

**Methods:**

We used semantic network analyses to categorize the reasons participants provided for joining a community during the sign-up process.

**Results:**

The most frequent reasons for joining were to seek information about their health condition, to help with self-management of health difficulties, for feelings of informational and social support, and for having a community with whom to share. Women were more likely to go online for sharing and companionship as well as for information and social support reasons, whereas men were more likely to go online for general information and self-management reasons.

**Conclusions:**

This study shows the reasons older adults seek to join a diabetes online community: for increased information and support regarding chronic health difficulties. Practitioners may want to consider ways to promote access to online communities among their older patients as a source of health information and a resource to provide a sense of community.

## Introduction

### Background

Across one’s lifespan, social interactions with same-aged peers—who are more likely to share attitudes, values, and interests—are important [1]. But barriers to mobility resulting from health difficulties may limit social contact [2,3]. Online communities, defined as collectives of voluntary members who share common interests or experiences and who interact primarily over the internet [4], may offer older adults, or people over age 65, an opportunity to engage with peers regardless of physical ability and location [3]. Online communities specifically for older adults are steadily growing [3], as increasing numbers of older adults have broadband access, use mobile phones, and are actively increasing their use of the internet [5] at the fastest rate of any population [6]. The potential for older adults to benefit from the internet for health information seeking [7,8], managing chronic conditions [7,9,10], interacting with similar peers [3], and engaging in online communities is starting to be recognized [11,12]. However, less attention has been paid to understanding older adults’ perceived benefits of joining online health communities, and few studies have directly examined older adults’ reasons for joining online communities.

In this study, we examined the reasons that older adults provided for joining an online community during their initial registration, to shed light on their needs and goals. The socioemotional selectivity theory suggests that older adults, due to perceived limitations on time and energy, are more likely to invest time *maintaining* quality social connections and balancing health states relative to forming *new* relationships and seeking *new* information [13]. However, it is unclear whether this distinction holds for online communication that removes physical barriers, potentially making it easier to interact, and for topics that are highly self-relevant such as those pertaining to one’s health [14]. Thus, older adults’ motivation for joining online communities may involve both forming new relationships and seeking information. Previous studies have shown that online communities may provide a space for older adults to seek health information, self-management strategies, and peer support and interaction [3,15,16]. We add to this literature by identifying reasons that older adults join online communities.

### Health Information Seeking

Although older adults receive health information from their primary care providers, seeking supplementary health information is still one of the most popular online activities [6], especially if the information given by health professionals is difficult to understand [9]. Some members of online communities report health care providers as the primary source of information [17], but participation in online communities can supplement that information through observing and interacting with individuals who have similar health conditions [9]. Older adults may find that health information in online communities differs from that of general websites because the information shared in online communities is often tailored to the unique needs of the group and the information may be more acceptable to receive from people with similar needs or goals [15]. The information may also be easier to understand, based on their social connections’ recent experience, and is readily available content [14].

Moreover, an online environment allows older adults to send and receive information to and from others asynchronously, thus reducing any restriction on time and mobility for receiving information about their condition [14], which may increase a sense of control [18]. In light of these findings, which suggest gathering general information regarding chronic health conditions is a key reason that older adults go online [19-22], we expect to find that one reason older adults join online health communities is to obtain general information.

### Self-Regulation and Management

More specifically, however, older adults likely join online communities to seek information related to self-management of a chronic health condition, which has been shown to contribute to older adults’ quality of life [21,23-25]. During times when primary providers are unavailable, older adults may need guidance in self-management of their health condition and may turn to the online community to receive that support [3,9]. People tend to trust others with shared experiences; the information shared in online communities may positively influence health behaviors [9]. One example would be community members co-constructing health knowledge and working together to fill gaps in health information to better understand their condition [3,26]. Therefore, we expect that individuals search for online communities to seek self-management information.

### Peer and Social Support

In addition to being a valuable resource for seeking health information, social support for chronic health difficulties may be another reason for joining online communities [27]. Receiving social support is particularly important for an individual’s well-being, by reducing stress and increasing adherence to treatment plans [15]. Low social connectedness is consistently associated with poorer health outcomes [28,29]. Those interacting in online communities may have more assistance in monitoring their condition and have a greater pool of self-management support resources [30,31].

Social support is especially valued when it comes from individuals with similar experiences [32]. A crucial benefit of online communities is that self-disclosure about chronic health conditions is perceived to be easier than in face-to-face discussion [3]. Allowing one to see their experiences as normal and receive praise for successful self-management, as well the confidence boost to reveal certain experiences to their provider is also an advantage [14]. In fact, greater social involvement online may lead to better self-management, physical health, and emotional well-being [9,28]. Although participation in online communities may not cure chronic health difficulties, the support from social connections may help improve the quality of life for older adults [27], thus it is essential to thoroughly understand the types of support being shared and received in online health communities. As previous studies have shown, individuals often go online to receive support for the information received from providers [19,21,22,33] and to receive social support to reduce adjustment difficulties that often coincide with chronic health difficulties [34-38]. Therefore, we expect individuals going to online communities for support will identify and cite reasons related to (1) information seeking and (2) maintaining contact with similar peers.

In this study, we investigated three general areas that older adults may offer as reasons they joined an online health community: (1) health information seeking, (2) self-regulation, and (3) social support. We drew on data from one of the largest diabetes online communities in the United States, the Diabetes Hands Foundation (DHF). The DHF was a nonprofit organization that “connects, engages and empowers people touched by diabetes.” At this time, the DHF has resolved and TuDiabetes is now part of the *Beyond Type I* organization. Leaders of DHF provided a de-identified dataset of the initial registration information collected when a new member joined the English-speaking language community (TuDiabetes.org). Based on literature reviewed above, we used semantic network analyses to confirm and further refine the reasons given for joining diabetes online communities in the areas of information-seeking, advice on self-management, and maintaining peer connections and receiving support from peers.

## Methods

The dataset included limited demographic information including age, sex, and diabetes type (I or II). The reason for joining was obtained from an open-ended question, “Why did you want to join?” Data for this study were obtained between June 12, 2007, and September 1, 2014, after which TuDiabetes began using a new database and no longer asked this question on joining. The dataset was retrieved in December 2014. Permission for this study was obtained from the Institutional Review Board at Northwestern University.

### Inclusion Criteria

Inclusion criteria for the study were that members had to be at least 65 years old and have type II diabetes. Age was reported by members at sign-up. The database contained 34,797 records: 30,248 participants were younger than 65 years old, 435 had type I diabetes, 49 had pre/no diabetes, and 2096 did not specify their age. The final sample included 1969 individuals, aged 65 and over, with type II diabetes.

### Ethical Approval and Consent

All procedures performed in studies involving human participants were in accordance with the ethical standards of the institutional and/or national research committee and with the 1964 Helsinki declaration and its later amendments or comparable ethical standards.

Consent was obtained from DHF for all participants included in the study.

### Procedure

We analyzed the unstructured free-text field responses that members provided on joining, in two phases.

#### Phase I

First, we examined the content of each response using semantic network analysis, which assesses the frequency of word co-occurrences [39]. The more frequently that two words co-occur, the more strongly they are related (as reflected in the pair’s “weight”). Centrality of a word, or the number of connections any word has with all other words, was also measured to reveal the importance of a concept in the dataset used (“weighted degree”). This approach has the added benefit of allowing us to produce a visual representation of the relationships among the concepts. We used ConText, which was created to conduct text and network analysis in an automated fashion for researchers in the digital humanities and social sciences [40], to construct the semantic network matrices, using the top 100 word pairs (co-occurrences of the words). To test intercoder reliability, a subsample of at least 10% of the full sample is required to be coded independently by independent coders [41]. In this study, a subsample of the top 26.52% word pairs (weight of 7077/26,685), or the top 100 word pairs, each with a weight of 25 or more, were coded by 3 independent coders. A weight below 25 meant that the word pair had occurred less than 0.10% (25/26,685) of the time, rendering those word pairs less significant. We then imported the top 100 word pairs into Gephi, a software for graph and network analysis that displays large networks for interactive exploration [42] and UCINET, which is used for graphical representation of network analysis [43] to run the matrix files in order to display the graphs and calculate each word’s centrality. This provided us with information on the connections among concepts within each open-ended response, and therefore, we referred to this as an item analysis.

**Figure 1:**
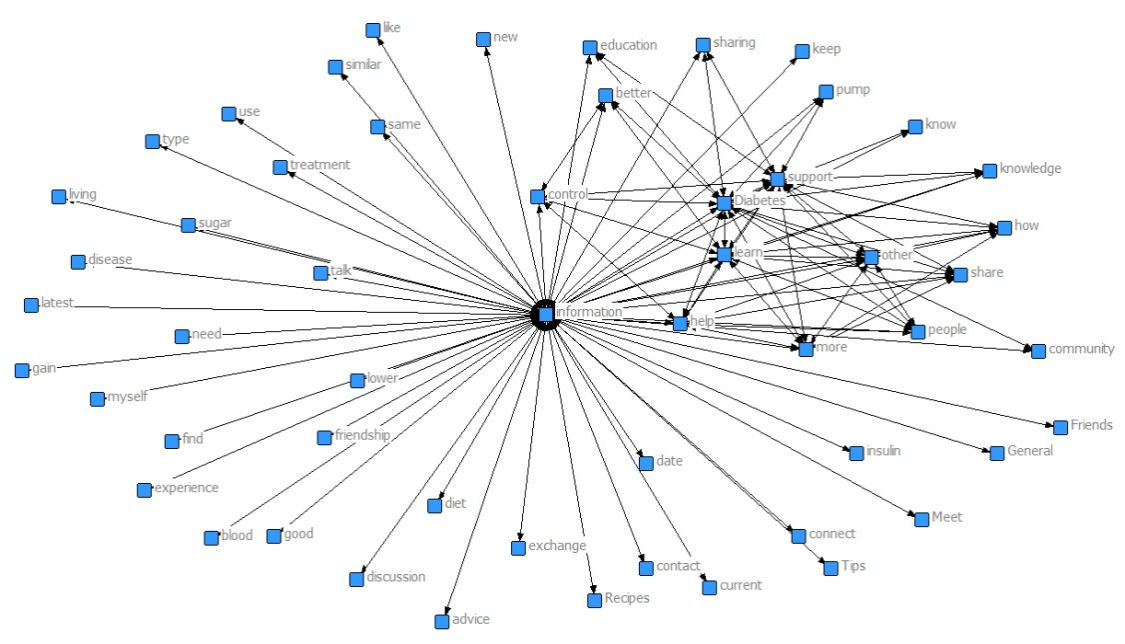
Degree of centrality of the words. This figure illustrates the relationship among the top 100 pairs: the more centered the words, the more significant they are.

**Figure 2:**
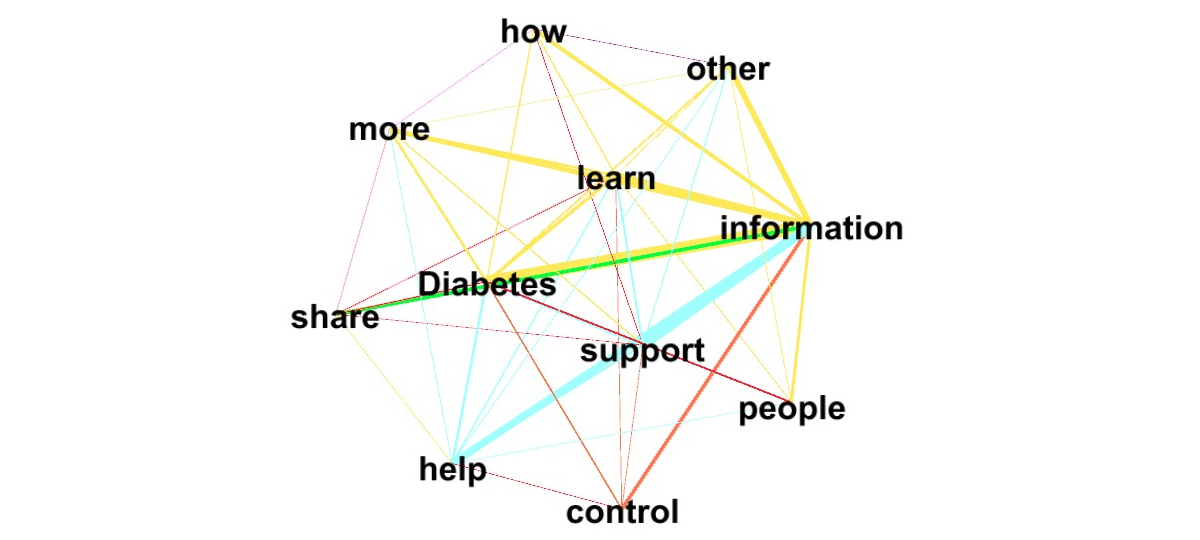
The strength of relationship between the top 15 word-pairs (the edges): the thicker the lines, the stronger the relationship for those word-pairs.

The output of the network analysis can be seen in Figure 1 (for all pairs) and Figure 2 (top weights only). The strength of the relationship between word pairs is denoted by line thickness. For example, the word pair “Diabetes information” (n=485) co-occurred most frequently, signified by the thickest line in Figure 2. Each of the top 12 word pairs (diabetes-information, support-information, help-information, learn-information, other-information, more-information, share-information, how-information, control-information, knowledge-information, learn-diabetes, and information-sharing) were related to sharing of information, giving an aggregated weight of 2762/7077, which is approximately 39.02% of the top 100 pairs.

#### Phase II

Borrowing the approach used by Wang et al [44] and taking into account the output from the semantic network analysis (Phase I), we identified broad categories into which the word pairs could be coded. We established the broad categories to provide an orienting framework to organize the word pairs, in order to have a way to consistently categorize the common ways that individuals use the DHF. We coded pairs (Table 1) as general information, self-management, share/support/companionship, informational support, and social support. Pairs were coded as:

- general information if they indicated that the new member sought advice, referrals, or knowledge [19,21,33]
- self-management if the word pairs indicated older adults going to the DHF for help with activities such as diet, self-regulation, pump, or medicine [23-25]
- share/support/companionship if the word pairs indicated anything involving two or more people and did not include words such as support, help, or advice [25,34,35]
- informational support if the word pairs were informational in nature and included words such as support, help, or advice without mentioning another person [21,22]
- social support if the word pairs were social in nature and included words such as support, help, or advice while mentioning another person [34,35,45]

We coded the top 100 word pairs (a weight of 7077/26,685) to determine their relative frequency. Word pairs were coded independently by 2 raters, yielding adequate reliability (Cohen’s kappa = .73). A third rater resolved disagreements.

In sum, we conducted two sets of analyses on these words used by older adults: item-level and person-level. Item-level analyses were conducted to assess the frequency of word co-occurrences. Person-level analyses were conducted to examine possible individuals’ differences in reasons for joining the DHF. Word pairs were always coded into the most specific categories if possible (self-management, share/support/companionship, informational support, social support). If word pairs could not be coded into the specific categories but were informational in nature, we coded them as general information. Less than 2% of the word pairs could not be coded into a category.

**Table 1:** Definitions of the coded categories and word pair examples.

Category	Definition	Actual word pair examples	References
Information	knowledge related	diabetes information; disease information; learn how	Bartlett & Coulson, 2011; Greene et al, 2010; Kaufman, 2010
Support (informational)	“support,” “help,” “advice,” without referencing another person	support how; information help	Menefee et al, 2016; Kaufman, 2010
Support (social)	“support,” “help,” “advice,” referencing another person	support people; help people	Crotty et al, 2015; Dale et al, 2012; Nicklett et al, 2013
Share/companionship	“share,” and involve other people	information community; share support; friendship information	Crotty et al, 2015; Strom & Egede, 2012; Vassilev et al, 2013
Self-management	diet, self-regulation, complication, blood, etc; pump and instrument; medicine	control better; treatment information; recipes information; pump information; diabetes pump; understand Super Bolus; insulin questions	Bodenheimer et al, 2002; Quinn et al, 2011; Vassilev et al, 2013
N/A	not applicable	other more; other how	

## Results

Item analyses (Table 2) showed that, among the most highly weighted 100 word pairs (ie, those having weight ≥25), 45.54% (3223/7077) reflected general information seeking. However, slightly more than half of the pairs fell into more specific categories. Specifically, 13.86% (981/7077) of the word pairs belonged to share/support/companionship, 16.83% (1191/7077) were categorized as expressing a desire for self-management (including medicine and pump), and 15.84% (1121/7077) and 5.94% (420/7077) indicated informational support and social support, respectively. Only 1.99% (141/7077) of the word pairs did not fall into one of our categories (see Table 2).

Person-level results are shown in Table 3. We found that 29.20% (574/1969 members) indicated that they joined the DHF to seek information but provided no additional information. On the other hand, the clear majority of new members provided information that could be more specifically coded. A large proportion of individuals, 18.10% (356/1969 members), stated that they joined the DHF for sharing/support/companionship purposes; 18.50% (365/1969 members) for information related to self-management (including pump and medication); 7.90% (155/1969 members) for informational “support” alone; 5.60% (111/1969 members) for social “support” alone; 3.65% (72/1969 visitors) gave responses that were not applicable; and 17.05% (336/1969 members) did not give an answer.

In addition to examining the percentage of older adults that endorsed the five categories or reasons for going online and interacting within the DHF, we also were interested in exploring whether older adult men and women in this sample differ in the rates that they endorse their respective reasons for joining the DHF. A chi-square test was run to determine whether men and women in this sample endorsed the reasons for joining the DHF at similar rates. Older adult men and women did not endorse each of the five reasons for joining the DHF at the same rates: χ^2^_4_=16.172 (N=1559), *P*=.003. As seen in Table 4, more older men than women in this sample endorsed the general information (41% men vs 33% women) and self-management categories (24% men vs 23%), whereas more older women than men endorsed the share/support/companionship (21% men vs 24% women), information support (8% men vs 12% women), and social support categories (6% men vs 8% women).

**Table 2:** Categories of word pairs (N=7077).

Category	n (%)
General information	3223 (45.54)
Other/Uncategorized	141 (1.99)
Self-management	1191 (16.83)
Share/Support/Companionship	981 (13.86)
Support (informational)	1121 (15.84)
Support (social)	420 (5.94)

**Table 3:** Reasons members gave for joining TuDiabetes (N=1969).

Category	n (%)
Health information seeking	575 (29.20)
Other/Uncategorized	408 (20.72)
Self-management	364 (18.50)
Share/Support/Companionship	110 (5.60)
Support (informational)	356 (18.10)
Support (social)	156 (7.92)

**Table 4:** Cross-tabulation of gender by category (N=1559).

Category	Gender^a^, n (%)	
	Men (n=789)	Women (n=770)
General information	323 (41.94)	251 (32.59)	
Self-management	186 (23.57)	179 (23.25)	
Share/support/companionship	168 (21.29)	188 (24.42)	
Information support	64 (8.11)	89 (11.56)	
Social support	48 (6.08)	63 (8.18)	

^a^χ^2^=16.172 (df=4); *P*<.01.

## Discussion

### Principal Considerations

Even with barriers to social contact in older age such as limitations on mobility as the result of health difficulties, online communities may be one way for individuals to have social contact regardless of time, location, or physical ability [2,3]. Engaging with peers online may be of particular importance as individuals, especially those experiencing chronic health difficulties, have an increasing need for information related to prevention, diagnosis, and treatment. Online communication may offer a viable option for dispersing information related to condition management [3,9]. In fact, the study’s results are in line with previous findings that older adults seek information online [6,14]. Not all individuals in this study who used the online community specified the type of information they sought. However, among those who did, there were several reasons specified: to seek information about their health condition, to help with self-management of health difficulties, for feelings of informational and social support, and for having a community with whom to share.

The results from this study add to the literature in a several ways. First, previous studies have shown that older adults do use online communities to obtain health information related to chronic conditions [6,14]. In order to understand diabetes patients’ use of online resources to seek health information, prior studies have frequently used interviews [46-48] and surveys [49,50]. Even in situations where they used content analysis, researchers performed only traditional quantitative content analysis [12]. Supplementing traditional quantitative content analysis with semantic network analysis as the current study did, allows for examination of users’ online information seeking behavior from a macro perspective. This method can reveal the relations among different words. In this study, we were able to more precisely specify, that for diabetes, older adults are motivated by the goal of obtaining information about medication and other self-management procedures. Additionally, previous studies have shown that having an online community of similar others may contribute to feelings of support for older adults [15,27]. Our findings add to the literature by showing that older adults hope to gain support, both informational and social. A particularly exciting finding is that the older adults in this sample indicate they are going to the online community for sharing and community purposes, suggesting that in addition to acting as a health information source, online communities may be one way that older adults are able to maintain feelings of community with similar others. Interestingly, it may be the case that older adult men and women endorse the reasons for going online at different rates. Our exploratory analyses show that men were more likely than women to provide reasons related to general information and self-management, whereas women were more likely to provide reasons related sharing, personal support, companionship, information support, and social support. These results may suggest that men are more likely to gather information to help manage a chronic health condition, while older adult women may be more likely to maintaining a sense of community or support while dealing with a chronic health condition. Future studies should further examine differences between older adult men and women, as they may be able to provide support to show consistent or systematic differences in the reasons that older adult men and women join online communities.

The results of this study do not appear to fully support the socioemotional selectivity theory [13], in that older adults in this study appeared to be motivated by obtaining information and by forming new social ties, rather than motivated by maintaining quality social connections and balancing health states. It could be that online health communities provide an exception to the theory because it is easier to form relations and gain information online without limitations on mobility and because health information is critical to well-being. It remains unclear, however, whether relations are maintained over time through these communities. A theory that might help to explain our results and that may be especially applicable when thinking about online communication or joining online communities is the Motivational Theory of Lifespan Development. This theory suggests that when individuals age, primary control, or the ability to influence environmental outcomes declines, increasing the need for secondary control strategies to maintain capacity for pursuing adaptive goals [51]. It could be that older adults’ use of online communities offers a new type of secondary control strategy for older adults with chronic illness, one that helps them maintain striving for their primary goals related to health and social contact.

### Future Directions

In general, the data show that both information and social support are key reasons why older adults join online health communities. More work is needed to examine the interactions between obtaining and using health information on the one hand and feeling socially connected to similar peers on the other. Past work has shown that high levels of engagement in diabetes online communities is associated with better glycemic levels, diabetes self-care, and health-related quality of life [52]. The role of peer relationships in online communities remains a key question for future research.

### Limitations

There are several limitations to our study. First, we relied on a naturalistic dataset with an open-ended question on reasons for joining that was likely interpreted differently across individuals and that did not provide an opportunity for follow-up questions when general responses were provided. In addition, we did not obtain information on continued use of the online community or on community members’ income or education levels, both of which are related to online use [3]. However, the goals of the study were not to examine continued use of the community, but rather to provide insight into the reasons why older adults joined a well-known online health community. With a basis from which to draw, in the future we will examine whether older adults continue to use the online community for these same reasons.

Additionally, we must consider the data reported here in light of the growth of social media use in recent years, that participants could have increasing alternatives for online communities. However, according to the Pew Research Center, Twitter use today remains very low among older adults (8%). While Facebook use is higher (41%), the majority of older adults do not use it for a specific purpose [53]. Although news feeds are a primary reason for using Facebook across ages, little is known about the likelihood of older adults’ use of Facebook for diabetes support. It is possible that growth in the memberships of other diabetes online communities could show similar patterns of reasons for joining as those reported in this study.

### Conclusions

Our findings suggest that older adults seek online communities for specific types of information regarding their chronic health conditions. As such, when designing an online community for use by older adults, it should be created so that it is easy for individuals to seek information from and share information with similar others, especially as it relates to medications and other self-management practices (technology tools). In addition, the results show that older adults seek online communities for social support. While older adults may be given sufficient health information from their primary care provider, they may find it useful to connect with similar others to better understand the information and how to apply it to their condition [9,16,17]. Thus, online communities should be designed with sharing and community purposes in mind, so that beyond being a site only for seeking information, the online community provides spaces for older adults to share personal stories, both success and struggles, and receive words of support from their peers who may understand them best [47].

## Abbreviations

DHF: Diabetes Hands Foundation

## References

[1] Ashida S, Heaney C (2008). Differential associations of social support and social connectedness with structural features of social networks and the health status of older adults. J Aging Health.

[2] Blieszner R (2014). The Worth of Friendship: Can Friends Keep Us Happy and Healthy. American Society on Aging.

[3] Leist A (2013). Social media use of older adults: a mini-review. Gerontology.

[4] Nimrod G (2013). Probing the audience of seniors' online communities. J Gerontol B Psychol Sci Soc Sci.

[5] Anderson M, Perrin A (2017). Tech adoption climbs amoung older adults.

[6] Magnezi R, Grosberg D, Novikov I, Ziv A, Shani M, Freedman L (2015). Characteristics of patients seeking health information online via social health networks versus general Internet sites: a comparative study. Inform Health Soc Care.

[7] Willis E (2016). Patients' self-efficacy within online health communities: facilitating chronic disease self-management behaviors through peer education. Health Commun.

[8] Smith A (2014). Older adults and technology use.

[9] Willis E (2014). The making of expert patients: the role of online health communities in arthritis self-management. J Health Psychol.

[10] Gee P, Greenwood D, Paterniti D, Ward D, Soederberg Miller LM (2015). The eHealth Enhanced Chronic Care Model: a theory derivation approach. J Med Internet Res.

[11] Chen Y, Schulz P (2016). The Effect of Information Communication Technology Interventions on Reducing Social Isolation in the Elderly: A Systematic Review. J Med Internet Res.

[12] Hilliard M, Sparling K, Hitchcock J, Oser T, Hood K (2015). The Emerging Diabetes Online Community. CDR.

[13] Carstensen L, Fung H, Charles S (2003). Socioemotional Selectivity Theory and the Regulation of Emotion in the Second Half of Life. Motivation and Emotion.

[14] Allen C, Vassilev I, Kennedy A, Rogers A (2016). Long-Term Condition Self-Management Support in Online Communities: A Meta-Synthesis of Qualitative Papers. J Med Internet Res.

[15] Kazmer M, Lustria M, Cortese J, Burnett G, Kim J, Ma J, Frost J (2014). Distributed knowledge in an online patient support community: Authority and discovery. J Assn Inf Sci Tec.

[16] Czaja S, Boot W, Charness N, Rogers W, Sharit J (2018). Improving Social Support for Older Adults Through Technology: Findings From the PRISM Randomized Controlled Trial. Gerontologist.

[17] Dolce M (2011). The Internet as a source of health information: experiences of cancer survivors and caregivers with healthcare providers. Oncol Nurs Forum.

[18] Angouri J, Sanderson T (2016). ‘You'll find lots of help here’ unpacking the function of an online Rheumatoid Arthritis (RA) forum. Language & Communication.

[19] Bartlett Y, Coulson N (2011). An investigation into the empowerment effects of using online support groups and how this affects health professional/patient communication. Patient Educ Couns.

[20] Greene J, Choudhry N, Kilabuk E, Shrank W (2011). Online social networking by patients with diabetes: a qualitative evaluation of communication with Facebook. J Gen Intern Med.

[21] Kaufman N (2010). Internet and information technology use in treatment of diabetes. Int J Clin Pract Suppl.

[22] Menefee H, Thompson M, Guterbock T, Williams I, Valdez R (2016). Mechanisms of Communicating Health Information Through Facebook: Implications for Consumer Health Information Technology Design. J Med Internet Res.

[23] Bodenheimer T (2002). Patient Self-management of Chronic Disease in Primary Care. JAMA.

[24] Quinn C, Royak-Schaler R, Lender D, Steinle N, Gadalla S, Zhan M (2011). Patient understanding of diabetes self-management: participatory decision-making in diabetes care. J Diabetes Sci Technol.

[25] Vassilev I, Rogers A, Blickem C, Brooks H, Kapadia D, Kennedy A, Sanders C, Kirk S, Reeves D (2013). Social networks, the 'work' and work force of chronic illness self-management: a survey analysis of personal communities. PLoS One.

[26] Gee P, Greenwood D, Kim K, Perez S, Staggers N, DeVon HA (2012). Exploration of the e-patient phenomenon in nursing informatics. Nurs Outlook.

[27] Loane S, D'Alessandro S (2013). Communication That Changes Lives: Social Support Within an Online Health Community for ALS. Communication Quarterly.

[28] Reeves D, Blickem C, Vassilev I, Brooks H, Kennedy A, Richardson G, Rogers A (2014). The contribution of social networks to the health and self-management of patients with long-term conditions: a longitudinal study. PLoS One.

[29] Heaney C, Israel B, Glanz K, Rimer BK, Lewis FM (2002). Social networks and social support. Health behavior and health education: Theory, research, and practice. 3rd ed.

[30] Cornwell B (2011). Independence through social networks: bridging potential among older women and men. J Gerontol B Psychol Sci Soc Sci.

[31] Bender J, Katz J, Ferris L, Jadad A (2013). What is the role of online support from the perspective of facilitators of face-to-face support groups? A multi-method study of the use of breast cancer online communities. Patient Educ Couns.

[32] Barak A, Boniel-Nissim M, Suler J (2008). Fostering empowerment in online support groups. Computers in Human Behavior.

[33] Greene A (2015). Patient commentary: social media provides patients with support, information, and friendship. BMJ.

[34] Crotty M, Henderson J, Ward P, Fuller J, Rogers A, Kralik D, Gregory S (2015). Analysis of social networks supporting the self-management of type 2 diabetes for people with mental illness. BMC Health Serv Res.

[35] Dale J, Williams S, Bowyer V (2012). What is the effect of peer support on diabetes outcomes in adults? A systematic review. Diabet Med.

[36] Nicklett E (2012). Sex, Health Behaviors and Social Support: Functional Decline among Older Diabetics. Am Med J.

[37] Stopford R, Winkley K, Ismail K (2013). Social support and glycemic control in type 2 diabetes: a systematic review of observational studies. Patient Educ Couns.

[38] Strom J, Egede L (2012). The impact of social support on outcomes in adult patients with type 2 diabetes: a systematic review. Curr Diab Rep.

[39] Doerfel M (1998). What constitutes semantic network analysis? A comparison of research and methodologies. Connections.

[40] Deisner J (2014). ConText: Software for the Integrated Analysis of Text Data and Network Data. https://convention2.allacademic.com/one/ica/ica14/.

[41] Skalski P, Neuendor KA, Cajigas JA, Neuendorf KA (2017). Content analysis in the interactive media age. Content Analysis Guidebook.

[42] Bastian M, Heymann S, Jacomy M (2009). Gephi: An Open Source Software for Exploring and Manipulating Networks.

[43] Borgatti S, Everett M, Freeman L (1992). UCINET for Windows: Software for Social Network Analysis. Connections.

[44] Wang YC, Kraut R, Levine J (2012). To stay or leave?: the relationship of emotional and informational support to commitment in online health support groups.

[45] Nicklett E, Heisler M, Spencer M, Rosland A (2013). Direct social support and long-term health among middle-aged and older adults with type 2 diabetes mellitus. J Gerontol B Psychol Sci Soc Sci.

[46] Brady E, Segar J, Sanders C (2017). Accessing support and empowerment online: The experiences of individuals with diabetes. Health Expect.

[47] Litchman M, Rothwell E, Edelman L (2018). The diabetes online community: Older adults supporting self-care through peer health. Patient Educ Couns.

[48] Newman MW, Lauterbach D, Munson S, Resnick P, Morris M (2011). “It's not that I don't have problems, I'm just not putting them on Facebook”: Challenges and opportunities in using online social networks for health.

[49] Bohanny W, Wu S, Liu C, Yeh S, Tsay S, Wang T (2013). Health literacy, self-efficacy, and self-care behaviors in patients with type 2 diabetes mellitus. J Am Assoc Nurse Pract.

[50] Giménez-Pérez G, Recasens A, Simó O, Aguas T, Suárez A, Vila M, Castells I (2016). Use of communication technologies by people with type 1 diabetes in the social networking era. A chance for improvement. Prim Care Diabetes.

[51] Heckhausen J, Wrosch C, Schulz R (2010). A motivational theory of life-span development. Psychol Rev.

[52] Litchman M (2015). A multiple method analysis of peer health in the diabetes online community. Dissertation.

[53] Smith A, Anderson M (2018). Pew Research Center.

